# Oxygen-Vacancy-Rich V_2_O_5_@NC Composite with Enhanced Zinc-Storage Performance for Aqueous Zinc-Ion Batteries

**DOI:** 10.3390/ma18225216

**Published:** 2025-11-18

**Authors:** Taoyun Zhou, Pingyuan Liang, Shilin Li, Yun Cheng, Xinyu Li

**Affiliations:** 1 School of Information, Hunan University of Humanities, Science and Technology, Loudi 417000, China; taoyun_2000@163.com (T.Z.);; 2 College of Physics and Electronic Information Engineering, Guilin University of Technology, Guilin 541004, China

**Keywords:** aqueous zinc-ion battery, vanadium oxide, V_2_O_5_, oxygen vacancies, three-dimensional confined structure

## Abstract

The practical application of vanadium-based cathode materials in aqueous zinc-ion batteries (AZIBs) is severely hindered by vanadium dissolution, low electronic conductivity, and sluggish reaction kinetics in aqueous electrolytes. In this work, a three-dimensional confined V_2_O_5_@ nitrogen-doped carbon (V_2_O_5_@NC) composite was rationally designed and constructed through a dual-regulation strategy combining oxygen-vacancy engineering and conductive network enhancement. In this architecture, the nitrogen-doped carbon framework provides a highly conductive network and robust structural support, while in situ carbonization induces the generation of oxygen vacancies within V_2_O_5_. These oxygen vacancies cause lattice distortion and expand the interlayer spacing, thereby accelerating Zn^2+^ diffusion and improving reaction kinetics. Benefiting from this synergistic effect, the V_2_O_5_@NC electrode exhibits an excellent specific capacity of 437 mAh g^−1^ at 0.1 A g^−1^ and maintains a remarkable 89.3% capacity retention after 2000 cycles at 3 A g^−1^, demonstrating outstanding rate performance and cycling stability. This study provides new insights and an effective design strategy for developing high-performance cathode materials for next-generation aqueous zinc-ion batteries.

## 1. Introduction

Lithium-ion batteries (LIBs) have dominated the energy storage market for portable electronics and electric vehicles owing to their high operating voltage, superior energy density, and long cycle life [1,2]. However, the scarcity of lithium resources, uneven geographical distribution, and the inherent flammability of organic electrolytes raise serious concerns about cost, sustainability, and safety, thereby restricting their large-scale deployment, particularly in stationary energy storage and grid-scale applications [3,4]. To meet the rapidly growing demand for low-cost, safe, and sustainable energy storage systems, the exploration of alternative battery chemistries based on earth-abundant elements has become an urgent priority.

Among the emerging candidates, aqueous zinc-ion batteries (AZIBs) have received significant attention due to the high theoretical capacity of the zinc anode (820 mAh g^−1^), low redox potential (−0.762 V vs. SHE), intrinsic safety, and environmental benignity of aqueous electrolytes [5,6]. In addition, zinc metal can be directly used as an anode without the need for complex fabrication or protective coatings, which simplifies cell configuration and reduces production cost. Despite these advantages, the strong electrostatic interactions between divalent Zn^2+^ ions and the host frameworks often lead to sluggish ion diffusion, severe polarization, and structural collapse during long-term cycling. Consequently, the development of advanced cathode materials with rapid charge-transfer kinetics, high capacity, and excellent structural reversibility is essential for realizing high-performance AZIBs.

Vanadium-based compounds have emerged as promising cathode candidates owing to their variable valence states (V^5+^/V^4+^/V^3+^), high theoretical capacity, and layered structure that can accommodate multivalent ion insertion [7]. Among them, vanadium pentoxide (V_2_O_5_) stands out for its open framework and large interlayer spacing, which enable reversible Zn^2+^ intercalation/deintercalation and confer high energy density [8]. Nevertheless, the large hydrated ionic radius of Zn^2+^ and its strong electrostatic interactions with oxygen atoms in the lattice can cause irreversible phase transitions, lattice collapse, and poor cycling stability during deep discharge.

To address these challenges, various strategies have been proposed. One common approach involves pre-intercalating cations such as Na^+^ [9], K^+^ [10], Ca^2+^ [11], and Mn^2+^ [12] into the V_2_O_5_ lattice. These guest ions act as structural “pillars”, effectively expanding the interlayer spacing and alleviating electrostatic repulsion between Zn^2+^ ions and the host framework, thereby enhancing structural stability and reversibility [13]. However, such pre-intercalated cations may gradually leach out during repeated cycling, leading to capacity degradation and loss of structural integrity.

An alternative and highly effective strategy to improve the electrochemical performance of V_2_O_5_ is to hybridize it with conductive carbonaceous materials such as graphene, carbon nanotubes, or amorphous nitrogen-doped carbon. The conductive carbon matrix not only buffers the volume expansion during ion insertion but also enhances electronic conductivity and electrode–electrolyte interfacial stability, thereby significantly improving reaction kinetics [14]. Nevertheless, even with carbon hybridization, the intrinsic sluggish Zn^2+^ diffusion and limited conversion reversibility of V_2_O_5_ remain unsolved.

Recently, oxygen-vacancy engineering has emerged as a particularly powerful approach to intrinsically enhance the electrochemical activity of V_2_O_5_. Oxygen vacancies (O_v) can effectively tailor the local electronic environment, lower the charge-transfer barrier, and introduce intermediate valence states (V^4+^), which collectively increase electrical conductivity and create additional Zn^2+^ adsorption sites [15,16,17]. For instance, Wang et al. [18] demonstrated that oxygen-deficient V_2_O_5_ nanosheets grown in situ on carbon cloth exhibited a high reversible capacity of 322 mAh g^−1^ at 1 A g^−1^—nearly twice that of pristine V_2_O_5_—highlighting the critical role of oxygen-vacancy modulation. Similarly, Zhang et al. [19] reported that oxygen-defective V_2_O_5_ nanobelts exhibited significantly improved Zn^2+^ diffusion kinetics, which they attributed to defect-induced electronic delocalization near the Fermi level. Moreover, Liu et al. [20] revealed through density functional theory (DFT) calculations that introducing oxygen vacancies effectively lowers the Zn^2+^ migration energy barrier and stabilizes the V_2_O_5_ lattice during repeated insertion and extraction. Collectively, these findings demonstrate that oxygen vacancies serve as crucial structural and electronic regulators that accelerate redox kinetics and improve long-term cycling stability in vanadium-based cathodes.

In addition to these direct oxygen-vacancy engineering strategies, several studies have explored indirect pathways to modulate the defect chemistry and electronic structure of V_2_O_5_-based cathodes. For example, Chen et al. [21] fabricated a V_3_O_7_/V_2_O_5_ composite, where partial oxygen loss occurred at the phase interface during synthesis, leading to increased V^4+^ content and enhanced Zn^2+^ diffusion kinetics. Similarly, Ding et al. [14] developed a porous V_2_O_3_@C composite, in which the metallic V_2_O_3_ phase inherently contained abundant oxygen vacancies, substantially improving both electrical conductivity and cycling durability. Du et al. [22] introduced conductive polyaniline (PANI) into V_2_O_5_ to form an organic–inorganic hybrid structure; the strong interfacial interactions between PANI and V_2_O_5_ partially reduced surface vanadium species, generating oxygen-deficient sites that promoted faster charge transfer. More recently, Li et al. [23] designed an in situ PANI-intercalated V_2_O_5_ architecture, in which the redox polymerization process expanded interlayer spacing and induced structural defects, further facilitating ionic transport and improving structural stability.

These studies collectively indicate that oxygen-vacancy formation—whether intentionally introduced or indirectly generated through compositional modulation, heterostructure construction, or polymer intercalation—plays a critical role in optimizing the electrochemical behavior of V_2_O_5_-based cathodes. Such defect-regulated mechanisms effectively balance electronic conductivity, Zn^2+^ diffusion kinetics, and structural robustness, offering valuable guidance for the rational design of next-generation high-performance aqueous Zn-ion batteries.

Beyond oxygen-vacancy modification, recent advances in doping engineering and heterostructure design have further improved the electrochemical properties of AZIB cathodes. Ji et al. [24] systematically reviewed the role of cation doping in MnO_x_-based materials, revealing that appropriate dopant incorporation can alleviate Jahn–Teller distortion, stabilize crystal frameworks, and enhance redox kinetics by optimizing electronic structures. Similarly, Zheng et al. [25] rationally designed a Ni-doped V_2_O_5_@3D Ni core–shell composite for high-voltage AZIBs, where Ni doping expanded the interlayer spacing of V_2_O_5_ and accelerated Zn^2+^ diffusion, while the three-dimensional metallic scaffold provided efficient electron-transport channels.

Building upon these insights, this work proposes a dual-engineering strategy that integrates oxygen-vacancy regulation with conductive network construction to simultaneously optimize both electronic and ionic transport in V_2_O_5_-based cathodes. Specifically, oxygen-vacancy-rich V_2_O_5_ nanoparticles are confined within a nitrogen-doped carbon (NC) matrix via an in situ polymerization–carbonization process, forming a robust three-dimensional V_2_O_5_@NC composite. In this design, the NC framework provides continuous electron pathways and structural reinforcement, while the carbonization process induces abundant oxygen vacancies in V_2_O_5_. These vacancies cause moderate lattice distortion and interlayer expansion, facilitating Zn^2+^ diffusion and improving the electrode’s structural integrity. Benefiting from the synergistic effects of defect engineering and conductive confinement, the resulting V_2_O_5_@NC composite delivers a high specific capacity of 437 mAh g^−1^ at 0.1 A g^−1^ and maintains 252 mAh g^−1^ even at 3 A g^−1^, with 89.3% capacity retention after 2000 cycles. This study offers a rational structural design concept for developing advanced, high-rate, and long-life cathode materials for next-generation aqueous zinc-ion batteries.

## 2. Materials and Methods

### 2.1. Materials and Characterization Instruments

Ammonium metavanadate (NH_4_VO_3_, analytical grade), dopamine hydrochloride (C_8_H_11_NO_2_·HCl, 99%), oxalic acid (H_2_C_2_O_4_, analytical grade), and methanol (CH_3_OH, analytical grade) were all purchased from commercial suppliers, including Shanghai Aladdin Biochemical Technology Co., Ltd. (Shanghai, China), Xilong Chemical Co., Ltd. (Shenzhen, China), and Xuzhou Xinnuo Chemical Co., Ltd. (Xuzhou, China), and used as received without further purification.

The morphology and microstructure of the samples were characterized by scanning electron microscopy (SEM, Hitachi S-4700, Hitachi Ltd., Tokyo, Japan)and transmission electron microscopy (TEM, JEM-2100F, JEOL Ltd., Tokyo, Japan). The crystal structure was analyzed using X-ray diffraction (XRD, Rigaku MiniFlex-600, Rigaku Corporation, Tokyo, Japan) with Cu Kα radiation (λ = 1.5406 Å). Raman spectra were collected on a Thermo Scientific DXR 2Xi system (Thermo Electron Scientific Instruments LLC, Madison, WI, USA) equipped with a 532 nm laser to evaluate the carbon structure. Thermogravimetric analysis (TGA) was carried out on a TA Instruments SDT Q600 (TA Instruments, New Castle, DE, USA) under a nitrogen atmosphere with a heating rate of 10 °C min^−1^.

Electrochemical measurements were performed using a CHI760E electrochemical workstation (CH Instruments, Shanghai, China).

### 2.2. Synthesis of V_2_O_5_ Nanoparticles (V_2_O_5_ NPs)

Typically, 1.0 g of NH_4_VO_3_ and 2.2 g of oxalic acid (H_2_C_2_O_4_) were dissolved separately in 60 mL of deionized (DI) water under continuous stirring at room temperature for 30 min. Subsequently, 0.4 mL of nitric acid (HNO_3_) was added to the mixed solution and stirred for an additional 5 min. The resulting homogeneous solution was then transferred into a 100 mL Teflon-lined stainless-steel autoclave and maintained at 190 °C for 5 h.

In this synthesis system, H_2_C_2_O_4_ serves as both a complexing and a mild reducing agent, which coordinates with vanadate ions (VO_3_^−^) to form soluble vanadyl–oxalate complexes and partially reduces V^5+^ to V^4+^. This controlled reduction helps regulate the nucleation and growth of vanadium oxide species during the hydrothermal process, yielding uniform nanosized precursors. Meanwhile, HNO_3_ acts as a pH regulator and oxidizing modulator, promoting the oxidation and stabilization of intermediate vanadium species, thereby facilitating the formation of phase-pure V_2_O_5_ with improved crystallinity after calcination.

After natural cooling to room temperature, the obtained blue-black precipitate was collected by centrifugation, washed several times with DI water and ethanol, and dried at 80 °C for 12 h. Finally, the dried product was calcined in air at 500 °C for 2 h to obtain crystalline V_2_O_5_ nanoparticles (V_2_O_5_ NPs).

### 2.3. Synthesis of V_2_O_5_@NC Composite

In a typical synthesis, 324 mg of V_2_O_5_ and 324 mg of dopamine hydrochloride (DA·HCl) were dispersed in a mixed solvent of methanol (120 mL) and deionized water (80 mL). The solution was stirred for 30 min at room temperature to ensure uniform dispersion. Then, 240 μL of ammonia solution (NH_3_·H_2_O) was added dropwise, and the mixture was stirred continuously at 50 °C for 24 h to induce the polymerization of dopamine on the V_2_O_5_ surface. The resulting precipitate was collected by centrifugation, washed repeatedly with ethanol and DI water, and dried at 60 °C overnight to obtain the V_2_O_5_@PDA intermediate.

Subsequently, the precursor was annealed at 650 °C for 2 h under a nitrogen atmosphere to carbonize the polydopamine layer, yielding the V_2_O_5_@NC composite.

### 2.4. Assembly of Aqueous Zinc-Ion Batteries

To fabricate the cathode, the active material (V_2_O_5_@NC), conductive carbon (Super P), and polyvinylidene fluoride (PVDF) binder were mixed in a mass ratio of 7:2:1 and dispersed in N-methyl-2-pyrrolidone (NMP) to form a uniform slurry. The slurry was coated onto a stainless steel mesh current collector, followed by drying at 60 °C for 12 h under vacuum to obtain the composite electrode.

The aqueous zinc-ion battery was assembled in a CR2016-type coin cell, using zinc foil as the anode, 3 M Zn(CF_3_SO_3_)_2_ aqueous solution as the electrolyte, and glass fiber membrane as the separator. All assembled cells were aged for 12 h under ambient conditions to allow sufficient electrolyte infiltration and interface stabilization. This mild resting process does not cause structural or compositional changes in the electrode materials but ensures consistent electrochemical performance [5].

## 3. Results and Analysis

### 3.1. Material Characterization

#### 3.1.1. SEM and TEM Characterization

The V_2_O_5_@NC composite with a three-dimensional confined structure was successfully synthesized via an in situ polymerization–carbonization strategy, in which V_2_O_5_ nanoparticles were encapsulated within a nitrogen-doped carbon framework. Initially, irregularly shaped V_2_O_5_ nanoparticles (V_2_O_5_ NPs) were obtained through a hydrothermal process followed by air annealing. The surface morphology and particle size of the as-prepared V_2_O_5_ and V_2_O_5_@NC samples were characterized by scanning electron microscopy (SEM, Hitachi S-4700, Hitachi Ltd., Tokyo, Japan) operated at an accelerating voltage of 10 kV, while the microstructure and lattice characteristics were further examined by transmission electron microscopy (TEM, JEM-2100F, JEOL Ltd., Tokyo, Japan) operated at an accelerating voltage of 200 kV.

**Figure 1:**
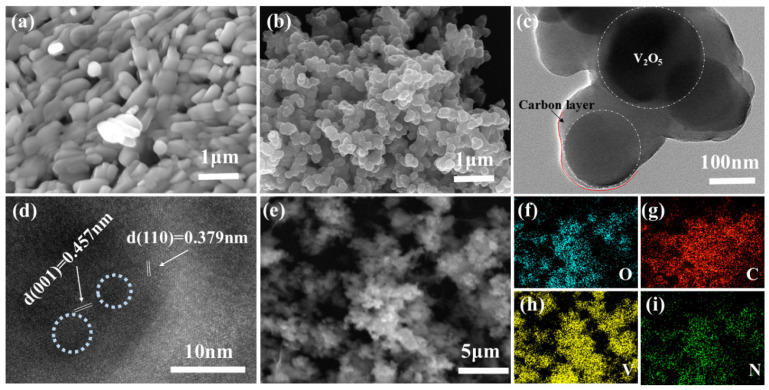
(**a**) SEM image of V_2_O_5_ NPs; (**b**) SEM image of the V_2_O_5_@NC; (**c**) TEM image of V_2_O_5_@NC; (**d**) HRTEM image of V_2_O_5_@NC; (**e**–**i**) EDS elemental mapping images of V, O, C, and N in the V_2_O_5_@NC composite.

The representative SEM and TEM images are shown in Figure 1, clearly illustrating the morphological evolution and structural configuration of the composite. As shown in Figure 1a, the SEM image reveals that the pristine V_2_O_5_ nanoparticles (V_2_O_5_ NPs) possess a rod-like morphology with an average diameter of approximately 500 nm. Subsequently, a uniform polydopamine (PDA) coating was achieved through the strong coordination interaction between the catechol groups of PDA and the surface vanadium species of V_2_O_5_. The polymerization was conducted in a 50 °C water bath for 24 h, which effectively balanced the nitrogen doping level and ensured the uniformity of the carbon precursor layer. The PDA served as both carbon and nitrogen sources during annealing at 650 °C under an inert atmosphere, resulting in the formation of nitrogen-doped carbon-coated V_2_O_5_ nanoparticles (V_2_O_5_@NC).

As shown in Figure 1b, compared with the pristine V_2_O_5_ rods, the V_2_O_5_@NC sample exhibits a more rounded and compact morphology, accompanied by a reduced average particle size of around 200 nm. This morphological transformation can be attributed to the synergistic effects of surface tension-driven reshaping during the carbonization process, partial lattice oxygen release, and confinement by the carbon shell, which collectively promote surface smoothing and particle densification. The carbon coating effectively inhibits grain overgrowth while maintaining nanoscale dimensions, leading to a narrower particle size distribution that facilitates bulk Zn^2+^ ion diffusion and enhances electrochemical kinetics [26].

The microstructure and crystalline characteristics of V_2_O_5_@NC were further investigated by TEM. As displayed in Figure 1c, a clear contrast difference between the V_2_O_5_ core and the surrounding carbon shell can be observed, with the coating layer thickness ranging from 20 to 50 nm. This well-defined core–shell configuration originates from the ordered molecular self-assembly of PDA under optimized polymerization conditions, forming a three-dimensional confined architecture in which V_2_O_5_ NPs are uniformly encapsulated by conductive carbon. Moreover, the carbon network is interconnected among nanoparticles, providing continuous electron pathways and facilitating electrolyte penetration, owing to the directional adsorption of PDA on the V_2_O_5_ surface [27].

As shown in Figure 1d, the HRTEM image of V_2_O_5_@NC exhibits clear lattice fringes with interplanar spacings of 0.457 nm and 0.379 nm, corresponding to the (001) and (110) planes of orthorhombic V_2_O_5_, respectively. Notably, several distorted lattice regions and defects (highlighted in light blue circles) indicate the presence of oxygen vacancies.

The well-defined lattice fringes confirm the high crystallinity of V_2_O_5_ and suggest the possible presence of oxygen vacancies, which can facilitate charge transport and ion diffusion. The corresponding STEM image and elemental mapping (Figure 1e–i) reveal the uniform distribution of V, O, C, and N throughout the composite, confirming the successful incorporation of nitrogen-doped carbon and the formation of oxygen-vacancy-rich V_2_O_5_@NC composite. Such a structure is expected to provide abundant active sites, enhance electronic conductivity, and improve the overall electrochemical performance of aqueous zinc-ion batteries.

#### 3.1.2. X-Ray Diffraction and Raman Spectroscopy Analysis

**Figure 2:**
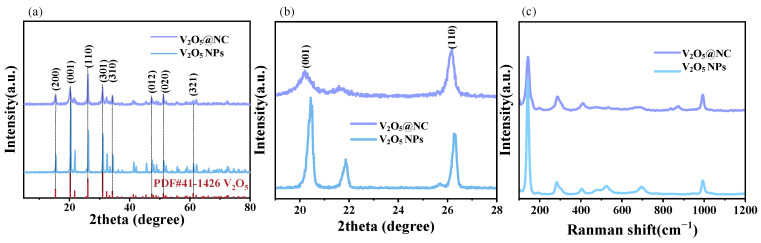
(**a**,**b**) XRD patterns of V_2_O_5_ NPs and the V_2_O_5_@NC composite; (**c**) Raman spectroscopy of the V_2_O_5_@NC composite.

X-ray diffraction (XRD) and Raman spectroscopy were employed to further elucidate the structural evolution and defect-regulation mechanism of the V_2_O_5_@NC composite. As shown in Figure 2a, both V_2_O_5_ NPs and V_2_O_5_@NC exhibit distinct diffraction peaks at 15.4°, 20.3°, 21.7°, 26.2°, 31.0°, 32.2°, and 34.3°, which can be indexed to the (200), (001), (101), (110), (310), (022), and (321) planes of orthorhombic V_2_O_5_ (JCPDS No. 41-1426), confirming that the main crystalline phase is V_2_O_5_.

In addition to the characteristic reflections of V_2_O_5_, several minor low-intensity peaks are observed in the XRD pattern of V_2_O_5_@NC. These peaks can be attributed to residual amorphous carbon species and trace vanadium suboxides (V_6_O_13_ or VO_2_) formed during the carbonization process under a mildly reductive atmosphere. Similar weak reflections have been reported for oxygen-deficient V_2_O_5_-based composites [28,29], indicating partial reduction of V^5+^ to V^4+^ associated with oxygen-vacancy generation rather than the formation of impurity phases. The absence of any strong additional diffraction peaks confirms that no secondary crystalline phases are present, and the samples can be regarded as phase-pure V_2_O_5_ with controlled defect modulation.

Moreover, compared with pristine V_2_O_5_ nanoparticles (V_2_O_5_ NPs), the diffraction peaks of V_2_O_5_@NC show a slight broadening and marginal shift toward lower 2θ angles, implying a reduced crystallite size and mild lattice expansion caused by oxygen-vacancy formation and interfacial strain from the nitrogen-doped carbon coating. Such structural modifications contribute to enhanced electronic conductivity and improved Zn^2+^ transport kinetics.

Notably, the (001) and (110) diffraction peaks of V_2_O_5_@NC shift toward lower angles by approximately 0.22° compared to pristine V_2_O_5_ NPs (Figure 2b), indicating an expansion of interplanar spacing. This lattice expansion can be attributed to the partial cleavage of V–O bonds during nitrogen doping, which induces the formation of oxygen vacancies and local lattice strain [13]. The enlarged interlayer spacing provides broader diffusion pathways for Zn^2+^ insertion/extraction, while the localized charge redistribution caused by oxygen vacancies effectively reduces the ion migration activation energy [30], thereby enhancing ion transport kinetics.

The Raman spectra (Figure 2c) display characteristic vibration modes of V_2_O_5_ at 286 cm^−1^ (O–V=O bending), 410 cm^−1^ (V–O–V symmetric stretching), 525 cm^−1^ (V–O–V asymmetric stretching), 696 cm^−1^, and 992 cm^−1^ (V=O stretching) [31]. Compared with pristine V_2_O_5_, the V_2_O_5_@NC composite exhibits an additional weak peak at around 875 cm^−1^, which can be ascribed to a defect-induced vibrational mode associated with oxygen vacancies [16]. This observation, consistent with the XRD-derived lattice expansion, provides further evidence for the successful introduction of oxygen defects.

Overall, the combined XRD and Raman analyses indicate that the nitrogen-doped carbon confinement not only stabilizes the V_2_O_5_ host lattice but also synergistically optimizes ion transport kinetics and structural robustness through lattice expansion and oxygen-vacancy engineering.

#### 3.1.3. X-Ray Photoelectron Spectroscopy and Thermogravimetric Analysis

**Figure 3:**
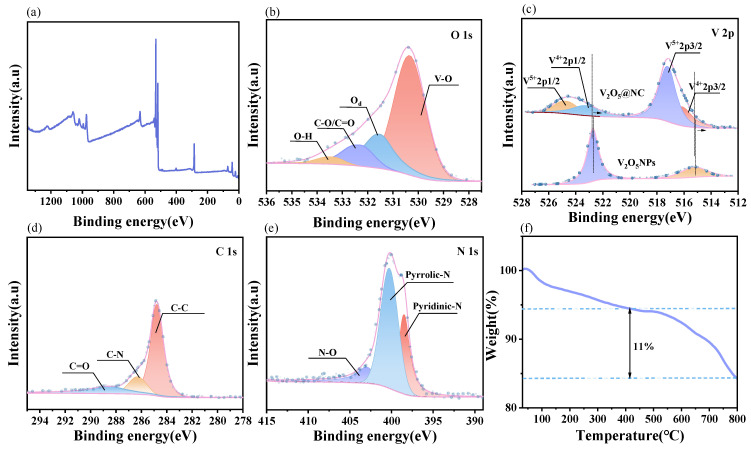
(**a**) XPS survey spectrum of the V_2_O_5_@NC composite; (**b**) High-resolution O 1s spectrum; (**c**) High-resolution V 2p spectrum; (**d**) High-resolution C 1s spectrum; (**e**) High-resolution N 1s spectrum; (**f**) TGA curve of the V_2_O_5_@NC composite.

X-ray photoelectron spectroscopy (XPS) and thermogravimetric analysis (TGA) were conducted to investigate the electronic structure, defect features, and compositional characteristics of the V_2_O_5_@NC composite. As shown in Figure 3a, the XPS survey spectrum exhibits distinct peaks corresponding to N 1s, V 2p, C 1s, and O 1s, confirming the formation of a 3D confined architecture composed of a V_2_O_5_ core and a nitrogen-doped carbon shell.

The O 1s spectrum (Figure 3b) can be deconvoluted into four peaks at 530.34, 531.51, 532.29, and 533.39 eV, assigned to V–O, oxygen vacancies (O_d), C–O/C=O, and O–H bonds, respectively. The distinct component at ~531.51 eV implies the presence of oxygen vacancies in V_2_O_5_@NC, which can improve electronic conductivity and provide additional active sites for Zn^2+^ storage. These results indicate that the mild polymerization temperature (50 °C) favors the maintenance of a locally reductive atmosphere during annealing, thereby promoting oxygen-vacancy formation via partial carbothermal reduction [15].

As shown in Figure 3c, the V 2p spectrum exhibits two pairs of peaks corresponding to V^5+^ (V 2p_3_/_2_ at 517.3 eV and V 2p_1_/_2_ at 524.8 eV) and V^4+^ (V 2p_3_/_2_ at 516.1 eV and V 2p_1_/_2_ at 523.5 eV). Compared with pristine V_2_O_5_ nanoparticles (V_2_O_5_ NPs), the V_2_O_5_@NC sample shows a slightly higher proportion of V^4+^ species, suggesting partial reduction of V^5+^ to V^4+^ induced by the carbon coating and nitrogen doping. This mixed-valence state is favorable for enhanced charge transfer and redox activity during Zn^2+^ insertion/extraction processes [20,32].

The C 1s spectrum (Figure 3d) is resolved into three peaks centered at 284.6, 285.8, and 288.4 eV, which can be assigned to C–C, C–N, and C=O bonds, respectively, confirming the presence of nitrogen-containing carbon. The N 1s spectrum (Figure 3e) reveals three types of nitrogen configurations: pyridinic-N (398.6 eV), pyrrolic-N (400.1 eV), and N–O (402.5 eV). These nitrogen species are known to contribute to defect generation and enhance interfacial interaction between carbon and V_2_O_5_, thereby promoting ion diffusion and improving electrical conductivity [19].

To further clarify the statement regarding the oxygen-vacancy concentration, the O 1s spectra of pristine V_2_O_5_ and V_2_O_5_@NC were quantitatively deconvoluted. The relative atomic percentages of different oxygen species are summarized in Table 1, which clearly demonstrates the higher proportion of oxygen vacancies in the V_2_O_5_@NC composite.

The thermogravimetric (TGA) curve (Figure 3f) reveals two distinct stages of weight loss. A minor mass decrease of approximately 3.3% occurs between 100 °C and 350 °C, which can be ascribed to the removal of physically adsorbed water and low-molecular-weight organics. A more pronounced weight loss of about 11% is observed above 400 °C, mainly resulting from the decomposition of incompletely carbonized oxygen-containing groups and defective edge carbon, accompanied by gas release and internal structural rearrangement. According to this analysis, the carbon component accounts for approximately 11 wt% of the total composite. This moderate carbon content provides effective spatial confinement to suppress excessive lattice expansion of V_2_O_5_, while maintaining sufficient pore channels for rapid Zn^2+^ ion diffusion.

In summary, the V_2_O_5_@NC composite exhibits a three-dimensional conductive framework and a significantly higher oxygen-vacancy concentration (15.3%) compared with pristine V_2_O_5_ (6.7%) (Table 1). These structural defects, together with the nitrogen-doped carbon shell, synergistically enhance electronic conductivity, facilitate Zn^2+^ diffusion, and lay a solid foundation for the superior electrochemical performance of V_2_O_5_@NC in aqueous zinc-ion batteries.

### 3.2. Electrochemical Performance of the V_2_O_5_@NC Electrode

To evaluate the electrochemical performance of the V_2_O_5_@NC cathode material for aqueous zinc-ion batteries (AZIBs), CR2016-type coin cells were assembled under ambient conditions, using V_2_O_5_@NC as the cathode, zinc foil as the anode, and a 3 M Zn(CF_3_SO_3_)_2_ aqueous solution as the electrolyte.

#### 3.2.1. Cyclic Voltammetry and Galvanostatic Charge–Discharge Tests

The electrochemical behavior of the V_2_O_5_@NC composite was further investigated and compared with V_2_O_5_ nanoparticles (V_2_O_5_ NPs) using a Zn//3 M Zn(CF_3_SO_3_)_2_//V_2_O_5_@NC aqueous zinc-ion battery system to evaluate its zinc storage performance.

**Figure 4:**
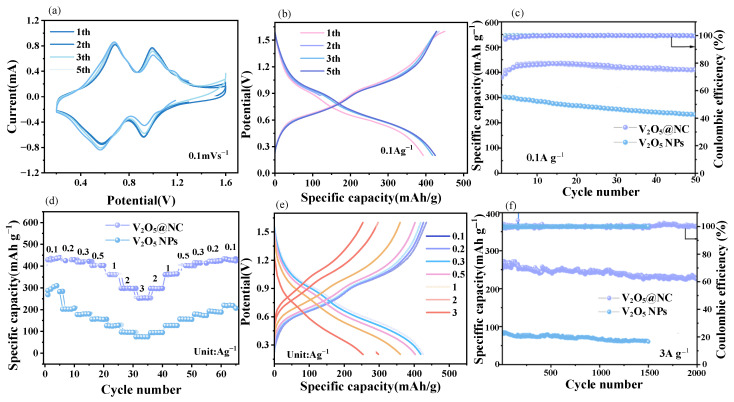
(**a**) CV curves of the V_2_O_5_@NC electrode at a scan rate of 0.1 mV s^−1^; (**b**) GCD curves of V_2_O_5_@NC at a current density of 0.1 A g^−1^; (**c**) Cycling performance of the V_2_O_5_@NC electrode at 0.1 A g^−1^; (**d**) Rate capability under various current densities; (**e**) Corresponding GCD curves under different current densities; (**f**) Long-term cycling stability at a high current density of 3 A g^−1^.

As shown in Figure 4a, the cyclic voltammetry (CV) curves were recorded in the potential range of 0.2–1.6 V at a scan rate of 0.1 mV s^−1^. Two pairs of well-defined redox peaks centered at 0.82/0.57 V and 0.91/0.77 V are observed, corresponding to the multi-step redox transitions of V^3+^/V^4+^ and V^4+^/V^5+^, respectively. The nearly overlapping CV profiles upon repeated cycling indicate a highly reversible Zn^2+^ insertion/extraction process within the V_2_O_5_@NC electrode.

**Figure 5:**
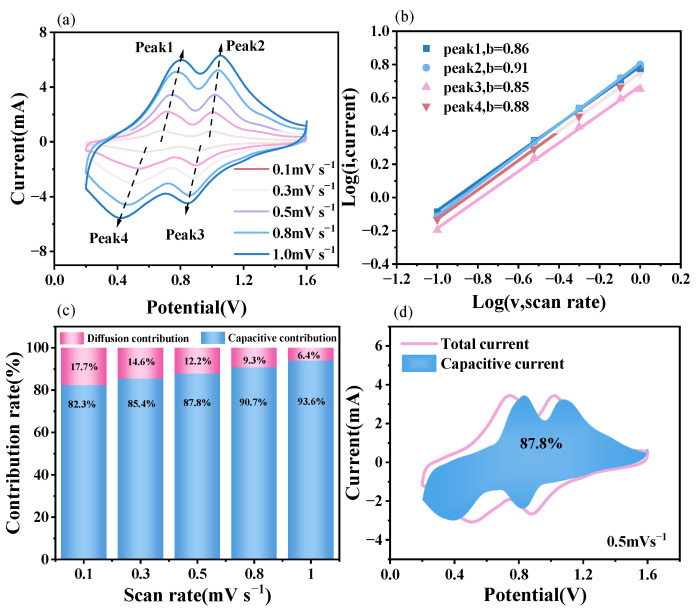
(**a**) CV curves of the V_2_O_5_@NC electrode recorded at different scan rates ranging from 0.1 to 1.0 mV s^−1^; (**b**) The corresponding log(*i*)–log(*v*) plots of the redox peaks; (**c**) Capacitive and diffusion-controlled contribution ratios at various scan rates; (**d**) Capacitive contribution at a scan rate of 0.5 mV s^−1^.

As shown in Figure 4b, the galvanostatic charge–discharge (GCD) profiles of V_2_O_5_@NC exhibit well-defined plateaus at approximately 0.89 V and 1.01 V, which correspond closely to the CV redox peaks, confirming the high reversibility of Zn^2+^ storage reactions. At 0.1 A g^−1^, the V_2_O_5_@NC electrode maintains a nearly stable specific capacity over 50 cycles with a Coulombic efficiency close to 100% (Figure 5c). Whereas the V_2_O_5_@NC electrode delivers an impressive specific capacity of 436 mAh g^−1^ after electrochemical activation, which is significantly higher than that of V_2_O_5_ NPs (301 mAh g^−1^), which show a gradual capacity decay. This enhanced capacity is primarily attributed to the three-dimensional conductive carbon framework that facilitates electron transport and the abundant oxygen vacancies that promote Zn^2+^ diffusion kinetics. Furthermore, after 50 cycles at 0.1 A g^−1^, V_2_O_5_@NC maintains a reversible capacity of 408 mAh g^−1^ with a high capacity retention of 93.5%, demonstrating excellent cycling durability.

The rate performance (Figure 4d) reveals that the V_2_O_5_@NC exhibits outstanding capacity retention across a wide range of current densities. The specific discharge capacities at 0.1, 0.2, 0.3, 0.5, 1, 2, and 3 A g^−1^ are 436, 428, 421, 402, 360, 297, and 252 mAh g^−1^, respectively, retaining 61.5% of its initial capacity when the current density increases from 0.1 to 3 A g^−1^. When the current density is reverted to 0.1 A g^−1^, the capacity recovers to 431 mAh g^−1^, corresponding to a high recovery rate of 98.8%, indicating excellent reversibility and structural robustness.

As illustrated in Figure 4e, the discharge plateau decreases only slightly from 0.91 V to 0.73 V as the current density increases from 0.1 to 3 A g^−1^, suggesting low polarization and efficient Zn^2+^ diffusion even at high rates. The nearly identical charge–discharge profiles under various current densities further validate the fast Zn^2+^ diffusion and outstanding electronic conductivity of the composite electrode. The unique architecture of V_2_O_5_@NC—where conductive N-doped carbon uniformly encapsulates V_2_O_5_ nanoparticles—creates efficient ion/electron transport channels and suppresses structural collapse during high-rate cycling.

At a high current density of 3 A g^−1^, V_2_O_5_@NC exhibits excellent cycling stability, retaining 94% of its initial capacity after 1500 cycles (Figure 4f). In contrast, V_2_O_5_ NPs suffer rapid capacity fading, mainly due to structural pulverization and sluggish reaction kinetics. After 2000 continuous cycles, the V_2_O_5_@NC electrode retains a high discharge capacity of 226 mAh g^−1^, corresponding to 89.3% capacity retention, with nearly 100% Coulombic efficiency.

These results demonstrate that the oxygen-vacancy-rich and nitrogen-doped carbon-confined V_2_O_5_ structure endows the electrode with superior reversibility, fast reaction kinetics, and remarkable structural stability during long-term cycling.

#### 3.2.2. Electrochemical Kinetic Analysis

To further investigate the Zn^2+^ storage kinetics of the V_2_O_5_@NC electrode, cyclic voltammetry (CV) measurements were performed at scan rates ranging from 0.1 to 1.0 mV s^−1^. As shown in Figure 5a, the CV curves of V_2_O_5_@NC display two pairs of distinct redox peaks, corresponding to the multistep valence transitions between V^3+^/V^4+^ and V^4+^/V^5+^.

As the scan rate increases, the peak currents increase proportionally, and the redox potentials shift slightly, indicating the coexistence of both surface-controlled and diffusion-controlled processes.

The relationship between the peak current (*i*) and the scan rate (*v*) follows the power-law equation:

*i* = *a*·*v^b^*, (1)

where *a* and *b* are adjustable parameters. The *b*-value, obtained from the slope of the log(*i*)–log(*v*) plot, can be used to determine the charge storage mechanism [33]. A *b*-value close to 0.5 indicates a diffusion-controlled process, whereas a *b*-value approaching 1 suggests a surface capacitive-dominated behavior [34]. As shown in Figure 5b, the calculated *b*-values of the redox peaks for the V_2_O_5_@NC electrode are 0.86, 0.91, 0.85, and 0.88, respectively. These values approaching unity indicate that the charge storage behavior of V_2_O_5_@NC is predominantly surface capacitive, rather than diffusion-limited, suggesting fast Zn^2+^ insertion/extraction kinetics facilitated by the conductive carbon network.

Based on the current response separation theory, the total current (*i*) can be divided into two parts: the diffusion-controlled contribution and the capacitive contribution, which can be expressed as:

*i* = *k*_1_ *v*^1/2^ + *k*_2_
*v*, (2)

where *k*_1_ *v*^1/2^ and *k*_2_ *v* correspond to the diffusion-controlled and capacitive-controlled processes, respectively, the relative capacitive contribution at different scan rates can be quantitatively determined.

As shown in Figure 5c, the capacitive contribution ratios of the V_2_O_5_@NC electrode are calculated to be 82.3%, 85.4%, 87.8%, 90.7%, and 93.6% at scan rates of 0.1, 0.3, 0.5, 0.8, and 1.0 mV s^−1^, respectively, confirming that capacitive behavior dominates at higher scan rates. Figure 5d further illustrates that at a scan rate of 0.5 mV s^−1^, the capacitive contribution reaches 87.8%, which is highlighted by the blue-shaded area.

These results demonstrate that the V_2_O_5_@NC composite electrode exhibits pseudocapacitive-dominated Zn^2+^ storage, benefiting from abundant electroactive sites, a conductive 3D carbon network, and structural defects (oxygen vacancies) that synergistically promote fast ion transport and redox reactions.

#### 3.2.3. Electrochemical Impedance Spectroscopy (EIS) Analysis

**Figure 6:**
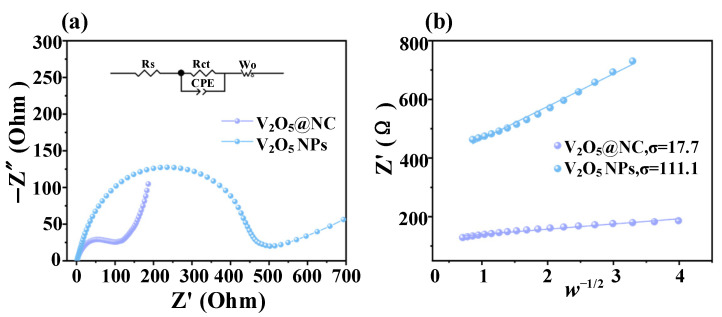
(**a**) Nyquist plots of the V_2_O_5_@NC and V_2_O_5_ NPs electrodes with the corresponding equivalent circuit model shown in the inset; (**b**) The linear relationship between the real impedance (Z′) and the square root of the reciprocal angular frequency (ω^−1/2^) in the low-frequency region.

To further investigate the charge-transfer characteristics, electrochemical impedance spectroscopy (EIS) measurements were conducted. As shown in Figure 6a, both electrodes exhibit typical Nyquist plots consisting of a semicircle in the high-to-medium frequency region and an inclined line in the low-frequency region, corresponding to the charge transfer process (Rct) and ion diffusion (Warburg impedance, Wₒ), respectively. The equivalent circuit (inset) includes Rs (solution resistance), Rct (charge transfer resistance), CPE (constant phase element), and Wₒ (Warburg element) for fitting. The V_2_O_5_@NC electrode shows a much smaller semicircle than pristine V_2_O_5_ NPs, indicating a substantially reduced charge transfer resistance (Rct = 94 Ω vs. 437 Ω). This demonstrates that the introduction of the nitrogen-doped carbon (NC) conductive framework effectively accelerates electron transport and improves the electrode–electrolyte interface kinetics.

Furthermore, the Zn^2+^ ion diffusion coefficient (D*_Zn_*) of the V_2_O_5_@NC electrode can be calculated from the Nyquist plots using the following equations:

*Z_real_* = *R_e_ + R_ct_ + σ w*^−1/2^, (3)

*D_zn_* = *R*^2^*T*^2^/2*A*^2^ *n*^4^ *F*^4^ *C*^2^*σ*
^2^, (4)

where ω is the angular frequency, *R* is the gas constant (8.314 J·K^−1^·mol^−1^), *T* is the absolute temperature in Kelvin (298.15 K for room temperature), *A* is the electrode surface area (2.01 cm^2^ for the 16 mm diameter electrode), *n* is the number of electrons transferred in the redox process (assumed to be 1 for the V^5+^/V^4+^ couple in this study), *F* is the Faraday constant (96,500 C·mol^−1^), and *C* is the Zn^2+^ ion concentration in the electrolyte (3.0 mol·L^−1^ = 3.0 × 10^−3^ mol·cm^−3^).

As shown in Figure 6b, the linear relationship between the real impedance (*Z*_real_, Z′) and ω^−1/2^ in the low-frequency region allows the determination of the Warburg coefficient (σ) from the slope of the fitted line. The σ values obtained from the fitting are 17.7 Ω·s^−1/2^ for V_2_O_5_@NC and 111.1 Ω·s^−1/2^ for V_2_O_5_ NPs, respectively. The much lower σ of V_2_O_5_@NC corresponds to a higher Zn^2+^ diffusion coefficient (D*_Zn_*), confirming that the synergistic effects of oxygen vacancies and the conductive NC network greatly facilitate Zn^2+^ transport.

To improve clarity and provide a comprehensive understanding of the EIS fitting results, we have included a summary of the key parameters used to calculate the Zn^2+^ ion diffusion coefficient (D*_Zn_*) in Table 2.

According to Equations (3) and (4), the calculated *D*_zn_ value of the V_2_O_5_@NC electrode is 9.93 × 10^−9^ cm^2^·s^−1^, which is nearly two orders of magnitude higher than that of pristine V_2_O_5_ NPs (9.97 × 10^−11^ cm^2^·s^−1^). This substantial improvement demonstrates that the nitrogen-doped carbon framework effectively accelerates Zn^2+^ ion diffusion and enhances charge-transfer efficiency, thereby improving the overall electrochemical performance of the electrode.

These findings are consistent with the GITT and CV results, indicating that the V_2_O_5_@NC composite possesses faster charge transfer kinetics and superior ionic conductivity, which are critical for achieving excellent rate capability and long-term cycling stability in aqueous Zn-ion batteries.

#### 3.2.4. Galvanostatic Intermittent Titration Technique (GITT) Analysis

**Figure 7:**
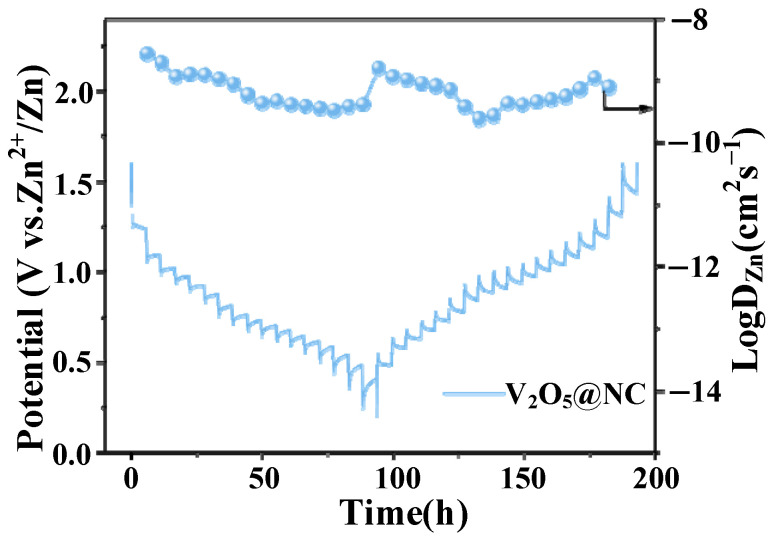
GITT curves and the corresponding calculated Zn^2+^ ion diffusion coefficients of the V_2_O_5_@NC electrode during the second discharge–charge process at a current density of 50 mA g^−1^.

In addition, to further investigate the influence of the enhanced electrical conductivity on Zn^2+^ ion diffusion kinetics, galvanostatic intermittent titration technique (GITT) measurements were carried out. As shown in Figure 7, the potential profile of the V_2_O_5_@NC electrode obtained from the GITT exhibits a series of quasi-equilibrium plateaus, which are consistent with the multistep redox behavior observed in the CV curves. The corresponding Zn^2+^ diffusion coefficients (D*_Zn_*), calculated based on Fick’s second law, fluctuate within the range of 2.69 × 10^−10^ to 2.63 × 10^−9^ cm^2^·s^−1^ during the charge–discharge process, which is consistent with the EIS analysis discussed earlier.

These results further confirm that the high electrical conductivity of the nitrogen-doped carbon framework, together with the presence of oxygen vacancies, significantly facilitates rapid Zn^2+^ ion transport and optimizes the electrode reaction kinetics. Consequently, the V_2_O_5_@NC electrode exhibits superior electrochemical performance with enhanced rate capability and cycling stability.

### 3.3. Comparative Performance Analysis of Vanadium-Based Cathodes for Aqueous Zn-Ion Batteries

To further highlight the electrochemical superiority of the oxygen-vacancy-rich V_2_O_5_@NC composite, its performance was systematically compared with representative vanadium-based cathode materials reported in the recent literature (Table 3) [14,21,22,23,35]. Various modification strategies—including heterostructure engineering, conductive polymer intercalation, and carbon hybridization—have been proposed to enhance Zn^2+^ storage kinetics, electronic conductivity, and cycling stability.

Chen et al. [21] developed a V_3_O_7_/V_2_O_5_ composite, where the synergistic redox interaction between V^3+^/V^4+^/V^5+^ facilitated efficient Zn^2+^ storage, yielding 176 mAh g^−1^ at 5 A g^−1^ and 96.2% capacity retention after 1120 cycles. Zhu et al. [35] investigated a hydrated VO_2_ (H-VO_2_) cathode with a synergistic H^+^/Zn^2+^ co-insertion mechanism, achieving 410 mAh g^−1^ at 0.1 A g^−1^ and 88% retention after 200 cycles, providing valuable insights into dual-ion transport behavior. Ding et al. [14] synthesized porous metallic V_2_O_3_@C, where the carbon framework enhanced electronic conductivity and structural robustness, resulting in 350 mAh g^−1^ at 0.1 A g^−1^, 250 mAh g^−1^ at 2 A g^−1^, and 90% retention after 4000 cycles. Du et al. [22] prepared an organic–inorganic V_2_O_5_@PANI hybrid, where the PANI coating enhanced interfacial contact and flexibility, providing 361 mAh g^−1^ at 0.1 A g^−1^ and 93.8% retention after 1000 cycles.

More recently, Li et al. [23] introduced an in situ PANI-intercalated V_2_O_5_ composite, where the conductive PANI chains expanded interlayer spacing and stabilized the structure, achieving 450 mAh g^−1^ at 0.1 A g^−1^ and 96.7% retention after 300 cycles at 1 A g^−1^, along with good rate performance up to 2 A g^−1^.

Although Li et al.’s hybrid electrode exhibited a slightly higher initial capacity, its cycling life was limited to 300 cycles. In contrast, the present V_2_O_5_@NC electrode maintains stable operation over 2000 cycles with minimal capacity degradation. Compared with systems relying solely on organic coatings or mixed-valence phase coupling, the dual-regulation strategy adopted here—combining oxygen-vacancy engineering and nitrogen-doped carbon confinement—provides a more efficient pathway to concurrently optimize electronic conductivity, Zn^2+^ diffusion kinetics, and structural durability.

Overall, the V_2_O_5_@NC composite developed in this study demonstrates superior comprehensive performance, with a high specific capacity of 437 mAh g^−1^ at 0.1 A g^−1^, an excellent rate capability of 252 mAh g^−1^ at 3 A g^−1^, and outstanding cycling stability (89.3% after 2000 cycles). These synergistic enhancements validate the effectiveness of integrating defect engineering with conductive framework design for constructing next-generation high-rate and long-life aqueous Zn-ion batteries.

## 4. Conclusions

In summary, a three-dimensional confined V_2_O_5_@NC composite material was successfully constructed via a dual-regulation strategy combining oxygen-vacancy engineering and conductive network reinforcement. Within this architecture, the nitrogen-doped carbon (NC) framework provides excellent electrical conductivity and structural stability, while in situ carbonization induces the formation of oxygen vacancies in V_2_O_5_ nanoparticles. These vacancies effectively optimize the crystal structure of V_2_O_5_ and broaden the ion diffusion channels, thereby significantly enhancing Zn^2+^ transport and insertion/extraction kinetics. Benefiting from the synergistic optimization of both electron transport and ion diffusion, the V_2_O_5_@NC composite electrode exhibits markedly improved zinc-storage performance compared to pristine V_2_O_5_. It delivers a high specific capacity of 437 mAh g^−1^ at 0.1 A g^−1^ and maintains 89.3% capacity retention after 2000 cycles at a high current density of 3 A g^−1^, demonstrating superior rate capability and cycling stability. This study highlights the effectiveness of integrating defect engineering with a conductive matrix to achieve enhanced electrochemical performance, offering a rational design strategy for developing next-generation Zn-ion battery cathode materials with high stability and rate performance.

## Figures and Tables

**Table 1:** Relative atomic percentage of oxygen species obtained from O 1s XPS peak deconvolution for pristine V_2_O_5_ and V_2_O_5_@NC composites.

Sample	V–O (Lattice Oxygen)	O_d_ (Oxygen Vacancy)	C–O/C=O	O–H	Total O (%)
Pristine V_2_O_5_	72.1%	6.7%	14.3%	6.9%	100
V_2_O_5_@NC composite	59.4%	15.3%	17.1%	8.2%	100

Note: The oxygen-vacancy (O_d_) fraction was determined from the deconvoluted O 1s peak centered at ~531.5 eV, corresponding to lattice-defect oxygen species. The O_d_ content in V_2_O_5_@NC (15.3%) is more than twice that of pristine V_2_O_5_ (6.7%), confirming that in situ carbonization under a mildly reductive environment effectively promotes oxygen-vacancy formation.

**Table 2:** Summary of EIS fitting parameters for V_2_O_5_@NC and V_2_O_5_ NPs electrodes.

Sample	Rs (Ω)	Rct (Ω)	CPE-T	CPE-P	Wo-R (Ω)	Wo-T (s)	Wo-P
V_2_O_5_@NC	12.5	94.0	3.12 × 10^−4^	0.89	98.5	8.6 × 10^−3^	0.71
V_2_O_5_ NPs	15.8	437.0	2.76 × 10^−4^	0.87	195.3	1.1 × 10^−2^	0.73

**Table 3:** Comparison of electrochemical performance of vanadium-based cathodes for aqueous Zn-ion batteries.

Ref.	Cathode Material	Modification Strategy	Specific Capacity	Rate Capability	Cycling Stability
This paper	V_2_O_5_@NC	Oxygen-vacancy & N-doped carbon dual regulation	437 @ 0.1 A g^−1^	252 @ 3 A g^−1^	89.3% after 2000 cycles
[21]	V_3_O_7_/V_2_O_5_	Synergistic phase interaction	176 @ 5 A g^−1^	high	96.2% after 1120 cycles
[35]	H-VO_2_	H^+^/Zn^2+^ dual-ion insertion	410 @ 0.1 A g^−1^	200 @ 5 A g^−1^	88% after 200 cycles
[14]	V_2_O_3_@C	Porous conductive carbon hybrid	350 @ 0.1 A g^−1^	250 @ 2 A g^−1^	90% after 4000 cycles
[22]	V_2_O_5_@PANI	Organic–inorganic composite	361 @ 0.1 A g^−1^	—	93.8% after 1000 cycles
[23]	PANI–V_2_O_5_ (in situ intercalated)	Interlayer expansion by PANI chains	450 @ 0.1 A g^−1^	220 @ 2 Ag^−1^	96.7% after 300 cycles@ 1 A g^−1^

## Data Availability

The original contributions presented in this study are included in the article. Further inquiries can be directed to the corresponding author.
